# Starch synthesis enzymes mediate oxidative stress-induced grain quality decline in rice under saline-alkali stress

**DOI:** 10.3389/fpls.2026.1955582

**Published:** 2026-10-19

**Authors:** Xiaotian Gu, Mengyan Sun, Yan Du, Guxi Gao, Ruchao Yin, Gongneng Feng, Bin Peng

**Affiliations:** 1 Yellow Sea Wetland Biological Resources and Green Intelligent Manufacturing Institute, Yancheng Institute of Technology, Yancheng, China; 2 Jiangsu Hufeng Grain & Oil Investment Development Co., Ltd., Yancheng, Jiangsu, China; 3 Funing County Agricultural Technology Extension Center, Funing, Jiangsu, China

**Keywords:** carbon–nitrogen metabolism, gelatinization, reactive oxygen species, saline-alkali stress, starch biosynthesis enzymes, starch crystalline structure

## Abstract

**Introduction:**

Soil salinization is a major constraint on rice production and grain quality, yet the physiological mechanisms linking saline-alkali stress to grain quality deterioration remain poorly understood.

**Methods:**

This study investigated the physiological cascade through which saline-alkali stress deteriorates grain quality, using four rice genotypes of contrasting salt tolerance as physiological models. Plants were grown under control and saline-alkali stress (3.0‰ soil salt) conditions, and grain quality, starch enzymes, oxidative stress markers, and starch structure were analyzed at key developmental stages.

**Results:**

Saline-alkali stress significantly inhibited AGPase, GBSS, SSS, and SBE activities across grain filling (15–45 DAP), with AGPase, SSS, and SBE showing the most pronounced suppression at 15 DAP, while GBSS exhibited a milder, genotype-dependent response. MDA accumulated markedly with genotype-dependent SOD/CAT imbalances. Correlation network analysis identified AGPase, SSS, and SBE as central hub nodes. XRD revealed reduced crystallinity; SEM showed altered granule morphology. Amylose increased while total starch decreased, reflecting carbon flux rerouting.

**Discussion:**

These findings delineate a physiological cascade of oxidative stress leading to enzyme inhibition, carbon-nitrogen imbalance, crystalline disruption, and quality deterioration, providing targets for genetic improvement. Future studies using ROS scavengers or gene editing are needed to establish causality.

## 1 Introduction

Soil salinization has emerged as one of the most pervasive and escalating abiotic constraints on global agricultural productivity, with recent assessments estimating that more than 1.4 billion hectares of land are now salt-affected worldwide (FAO, 2024). The continued expansion of secondary salinization, driven by irrational irrigation, climate change, and intensive cropping, is degrading arable land at an alarming rate, particularly in coastal and semi-arid regions where saline-alkali conditions prevail (FAO, 2024; Fang et al., 2021). Rice (Oryza sativa L.), a staple food for more than half of the global population, is classified as a moderately salt-sensitive cereal (Munns and Tester, 2008), and even moderate salinity can substantially compromise both grain yield and quality (van Zelm et al., 2020). Saline-alkali stress imposes a dual burden on plants. It combines osmotic stress, ionic toxicity, and high pH (Zhu, 2016), which collectively disrupt water uptake, nutrient homeostasis, and metabolic flux (van Zelm et al., 2020; Fang et al., 2021). With the global population projected to exceed 9.7 billion by 2050 and arable land per capita declining, understanding how rice responds to saline-alkali conditions is essential for safeguarding food security in vulnerable coastal agroecosystems (Ahmad et al., 2022).

Grain quality, rather than yield alone, has become a decisive determinant of the market value and consumer acceptance of rice, encompassing appearance, milling, eating and cooking, and nutritional attributes (Radha et al., 2023). Among these, amylose content, starch composition, protein accumulation, and pasting properties collectively define the cooking behavior, texture, and digestibility of the grain (Sangwongchai et al., 2022). Saline-alkali stress has been shown to perturb these quality traits, but the magnitude and direction of the changes are strongly genotype-dependent: salinity generally increases amylose content while reducing total starch, alters gel consistency, and modifies protein accumulation patterns (Sangwongchai et al., 2022; Li et al., 2023; Zhang et al., 2022; Alam et al., 2022). Recent studies have further demonstrated that salt stress at the reproductive stage can alter starch granule morphology, gelatinization behavior, and amylopectin fine structure in a cultivar-specific manner (Zhang et al., 2022; Sangwongchai et al., 2025; Alam et al., 2022). However, the reported effects vary considerably among cultivars and stress intensities, and the physiological basis for such variability remains incompletely characterized (Radha et al., 2023; Zhang et al., 2022). A systematic understanding of how saline-alkali stress reshapes the suite of grain quality traits across genotypes with contrasting salt tolerance is therefore urgently needed to inform the development of varieties that maintain high quality under marginal conditions.

At the physiological level, saline-alkali stress influences grain quality primarily by disrupting starch biosynthesis and triggering oxidative damage during grain filling. Starch accumulation in the developing endosperm depends on the coordinated activity of key enzymes, including ADP-glucose pyrophosphorylase (AGPase), granule-bound starch synthase (GBSS), soluble starch synthase (SSS), and starch branching enzyme (SBE), the expression and activity of which are sensitive to abiotic stress (Lee et al., 2021; Praphasanobol et al., 2025). Recent transcriptomic analyses have revealed that salt stress downregulates starch-synthesis-related genes, including GBSSI, SSS, and SBE, in developing seeds, with the extent of suppression correlating with starch physicochemical deterioration (Praphasanobol et al., 2025; Sangwongchai et al., 2025; Thongnok et al., 2024). Concurrently, the overproduction of reactive oxygen species (ROS) under salt stress leads to lipid peroxidation, membrane damage, and the perturbation of carbon and nitrogen metabolism (Tavu and Redillas, 2025; Du et al., 2025). The activation of antioxidant defenses, such as superoxide dismutase (SOD) and catalase (CAT), varies markedly among genotypes and contributes to differential stress tolerance (Tavu and Redillas, 2025; Ubaidillah et al., 2024). A growing body of evidence indicates that oxidative damage does not act independently but rather exerts its deleterious effects on grain quality largely through the inhibition of starch synthesis enzymes, highlighting the interplay between enzymatic starch synthesis, oxidative stress, and protein remodeling as a critical, yet underexplored, nexus governing saline-alkali-induced quality deterioration (Du et al., 2025; Sangwongchai et al., 2025; Jin et al., 2024).

Despite recent advances, several critical knowledge gaps persist. Most existing studies have examined individual quality traits or isolated physiological processes, and few have systematically compared multiple genotypes under realistic coastal saline-alkali conditions (Zhang et al., 2022; Wang et al., 2025). Moreover, the integration of grain quality parameters with starch synthesis enzyme activities and oxidative stress markers within a unified analytical framework remains limited, hindering a holistic understanding of the mechanisms linking stress perception to quality deterioration (Wang et al., 2025; Fernando et al., 2025). Specifically, it remains unclear whether oxidative stress during grain filling differentially inhibits starch synthesis enzymes in a genotype-dependent manner, and how this differential inhibition translates into the structural and physicochemical quality changes observed in rice grains under saline-alkali conditions. Consequently, the relative contributions of carbon-nitrogen imbalance, enzymatic inhibition, and oxidative damage to genotype-specific quality responses under saline-alkali stress remain poorly resolved, and an integrated mechanistic framework linking stress perception to quality deterioration is lacking (Wang et al., 2025; Fernando et al., 2025).

To address these gaps, the present study employed four rice varieties with contrasting salt tolerance (YD10, YD12, YD21, and NJ9108) as physiological models to investigate the mechanisms by which coastal saline-alkali stress (3.0‰ soil salt) in Jiangsu, China, compromises grain quality. The research strategy encompassed three interconnected layers: (i) phenotypic characterization of grain composition, starch physicochemical properties (RVA and DSC), protein fractions, and crystalline structure; (ii) mechanistic investigation of starch synthesis enzyme activities (AGPase, GBSS, SSS, SBE) and oxidative stress markers (MDA, SOD, CAT) during grain filling; and (iii) systems-level integration through correlation network analysis to identify key metabolic hubs linking oxidative stress to quality traits. We hypothesized that saline-alkali stress deteriorates grain quality through the coordinated inhibition of starch biosynthesis enzymes and the exacerbation of oxidative damage during grain filling, and that tolerant genotypes maintain superior quality by sustaining enzyme activity and antioxidant capacity. Specifically, we predicted that (i) oxidative stress would inhibit AGPase, SSS, and SBE more severely than GBSS, leading to a disproportionate reduction in amylopectin synthesis; (ii) this differential inhibition would disrupt starch crystalline structure and pasting properties; and (iii) salt-tolerant genotypes would maintain more stable enzyme activities and antioxidant capacity, thereby preserving grain quality. By integrating these complementary analytical dimensions, this study aims to (i) elucidate the physiological mechanisms underlying saline-alkali-induced quality deterioration, (ii) identify the key metabolic hubs linking oxidative stress to quality traits, and (iii) delineate the differential physiological strategies employed by tolerant versus sensitive genotypes, thereby providing a mechanistic framework for future targeted genetic improvement of rice quality under saline-alkali conditions.

## 2 Materials and methods

### 2.1 Plant materials and experimental design

Four rice varieties were used: Yandao 10, Yandao 12, Yandao 21, and Nanjing 9108. The first three were provided by the Institute of Agricultural Sciences of Coastal Area of Jiangsu, China, and are recognized as salt-tolerant germplasms based on preliminary field screening in coastal saline-alkali soils (soil salt content > 3.0‰), where these varieties consistently maintained >80% seed setting rate and stable yield (Li et al., 2023; Zhang et al., 2022). Nanjing 9108, supplied by the Institute of Food Crops, Jiangsu Academy of Agricultural Sciences, is a widely cultivated high-quality soft rice variety with excellent eating quality but high salt sensitivity, documented by its >40% yield reduction under moderate salinity in prior field trials (Zhang et al., 2022; Li et al., 2023).

The experiment was conducted at the Jianchuan Experimental Station, Dafeng District, Yancheng City, Jiangsu Province, China (33°20′ N, 120°43′ E), a typical coastal saline-alkali area along the Yellow Sea. The soil was chloride-type coastal saline soil with pH 8.6 ± 0.3 and a Na^+^/Cl^-^ ratio of 1.2 in the soluble salt fraction. Two treatments were established: control (CK), representing normal soil (salt content < 0.5‰, electrical conductivity [EC] = 0.8 dS/m) with freshwater irrigation; and saline-alkali stress (SS), representing soil with a total soluble salt content of 3.0‰ (EC = 4.8 dS/m). Each variety was planted in 3–5 rows with 30 cm row spacing and 15 cm plant spacing, with 50 cm isolation rows between plots and 2 m border rows. Sowing was performed on May 25, 2024, and transplanting on June 30, 2024. No fertilizer was applied to eliminate confounding effects of nutrient input on the stress response, particularly the influence of nitrogen on starch synthesis enzyme activities and protein accumulation. Weeds were removed manually.

### 2.2 Agronomic trait measurement and flour preparation

Plant height, effective tiller number, grains per panicle, 1000-grain weight, and seed setting rate were recorded at maturity from three representative panicles per plant. Grain yield per plant was calculated accordingly. Harvested grains were naturally dried to 13% moisture, dehulled using a SY88-TH husker (Korea), and polished to 90% milling degree using a VP-32T polisher (Yamamoto, Japan). The polished rice was ground into flour and passed through a 100-mesh sieve for physicochemical analyses.

### 2.3 Starch and soluble sugar analysis

Amylose content was determined by spectrophotometry (NY/T 2639-2014) at 620 nm using a UV-1800 spectrophotometer, with standard curves established from four reference rice flours. Total starch and soluble sugar contents were determined by the anthrone-sulfuric acid colorimetric method.

### 2.4 Protein analysis

Total protein was determined by the Kjeldahl method using an automatic analyzer (KJELTEC 8420, FOSS, Denmark), with a conversion factor of 5.95. Protein fractions—albumin, globulin, prolamin, and glutelin—were determined by the modified Bradford method (Sangon Biotech, China) at 595 nm using a Multiskan FC microplate reader.

### 2.5 Gel consistency

Gel consistency was measured following standard procedures. Rice flour was dispersed in KOH solution, heated, cooled, and the gel length was measured.

### 2.6 Pasting and thermal properties

Pasting properties were determined using a Rapid Visco Analyzer (RVA, Model 3D, Newport Science, Australia) following Batey and Curtin (2000). Rice flour (2.8 g) was suspended in 25.2 g ultrapure water (10% w/w), equilibrated at 50 °C for 1 min, heated to 95 °C at 12 °C/min, held for 2.5 min, cooled to 50 °C at 12 °C/min, and held for 1 min. Paddle speed was 960 rpm for the first 10 s and 160 rpm thereafter. Parameters recorded included peak viscosity, hot paste viscosity, breakdown, final viscosity, setback, pasting time, and pasting temperature.

Thermal properties were analyzed using a differential scanning calorimeter (DSC, Model 200 F3 Maia, Netzsch, Germany) following Lu and Lu (Lu and Lu, 2019). Samples (5 mg) were sealed in aluminum pans with 10 μL distilled water (66.7% moisture), equilibrated at 4 °C for 24 h, and heated from 20 °C to 100 °C at 10 °C/min. Onset temperature (T_o_), peak temperature (T_p_), conclusion temperature (Tc), and gelatinization enthalpy (ΔHgel) were recorded. After 7 days at 4 °C, retrogradation enthalpy (ΔHret) was measured, and retrogradation percentage was calculated as %R = 100 × ΔHret/ΔHgel.

### 2.7 Starch synthesis enzyme and oxidative stress marker assays

Key starch synthesis enzymes—ADP-glucose pyrophosphorylase (AGPase), granule-bound starch synthase (GBSS), soluble starch synthase (SSS), and starch branching enzyme (SBE)—were assayed in developing grains at 15, 30, and 45 days after pollination (DAP), representing early grain-filling/starch granule initiation (15 DAP), active starch accumulation (30 DAP), and late grain-filling/maturation (45 DAP) stages, using commercial assay kits (Comin Biotechnology, China) following the manufacturer’s protocols. Activities were expressed as U per gram fresh weight (U/g FW).

Oxidative stress markers were measured in flag leaves at the same time points. Malondialdehyde (MDA) content was determined by the thiobarbituric acid method. Superoxide dismutase (SOD) activity was assayed by the nitroblue tetrazolium method, and catalase (CAT) activity was measured by the ammonium molybdate method. All assays were performed with three biological replicates.

### 2.8 XRD and SEM analysis

Starch crystalline structure was analyzed by X-ray diffraction (XRD, D8 Advance, Bruker, Germany) using Cu Kα radiation at 40 kV and 40 mA. Samples were scanned from 3° to 50° 2θ at a speed of 2°/min. Relative crystallinity was calculated as the ratio of crystalline peak area to total diffraction area using Origin software.

Starch granule morphology was observed by scanning electron microscopy (SEM, SU8010, Hitachi, Japan). Rice flour was mounted on aluminum stubs with conductive tape, sputter-coated with gold, and examined at an accelerating voltage of 5.0 kV.

### 2.9 Statistical analysis

Data were analyzed using SPSS 23.0. Two-way ANOVA was performed with variety and salt treatment as fixed factors, and means were compared using Duncan’s multiple range test at *p < 0.05*. All measurements were performed with at least three biological replicates, and results are presented as mean ± standard deviation. Pearson correlation analysis was performed using R software (version 4.3.1), and correlation matrices were visualized with the corrplot package.

## 3 Results

### 3.1 Agronomic traits and yield components

Saline-alkali stress significantly suppressed agronomic traits and yield components in all varieties, with genotype-dependent responses (**Table 1**). Plant height decreased by 13–18% and effective tiller number by 4–10% under stress. Nanjing 9108 showed the largest yield reduction (48.1%), whereas Yandao 21 showed the smallest (24.4%), indicating stronger yield stability. Yield per plant, 1000-grain weight, and seed setting rate all showed genotype-dependent responses, with Yandao 21 maintaining the best overall performance.

**Table 1:** Agronomic traits of the four rice varieties under control (CK) and saline-alkali stress (SS).

Variety	Treatment	Plant height (cm)	Tiller no.	Grains per panicle	1000-grain weight (g)	Seed setting rate (%)	Yield per plant (g)
NJ9108	CK	79.92 d	9.67 b	123.30 a	23.81 b	97.77 a	28.40 a
NJ9108	SS	77.64 d	8.17 c	103.85 d	17.38 d	78.77 d	14.74 g
YD10	CK	97.15 a	9.29 b	114.85 b	20.96 c	95.12 a	22.36 c
YD10	SS	67.76 f	9.33 b	106.85 d	16.03 e	75.12 e	15.98 f
YD12	CK	89.12 b	8.33 c	110.85 c	25.12 a	94.98 a	23.19 c
YD12	SS	73.18 de	7.83 c	100.85 d	21.80 c	82.98 c	17.21 e
YD21	CK	89.18 b	9.40 b	109.35 c	24.67 a	95.42 a	25.35 b
YD21	SS	75.12 de	8.83 bc	110.89 c	19.61 cd	85.42 b	19.19 d

Values are means (n = 3). Different letters indicate significant differences at p < 0.05 (Duncan’s test).

### 3.2 Grain composition and quality parameters

Saline-alkali stress induced significant changes in grain composition, with clear genotypic differences (**Figure 1**). Amylose content increased significantly in all varieties (p < 0.01; **Figure 1A**), with Yandao 21 showing the greatest absolute increase (+1.92 mg/g) and Yandao 10 the greatest relative increase (+27.2%). Nanjing 9108 showed the smallest increase (+0.41 mg/g), yet its yield reduction was the largest, indicating that amylose stability alone does not confer overall salt tolerance. Calculation of the amylose-to-total starch ratio revealed that this ratio increased under stress in all varieties: in Yandao 10, the ratio increased from 0.127 to 0.162 (+27.2%) while total starch decreased from 78.8% to 76.0%; in Yandao 12, the ratio increased proportionally while total starch decreased from 70.0% to 69.0%. These data confirm that the amylose proportion increased because total starch (particularly amylopectin) declined more than absolute amylose content, providing quantitative support for the relative synthesis rate hypothesis.

**Figure 1:**
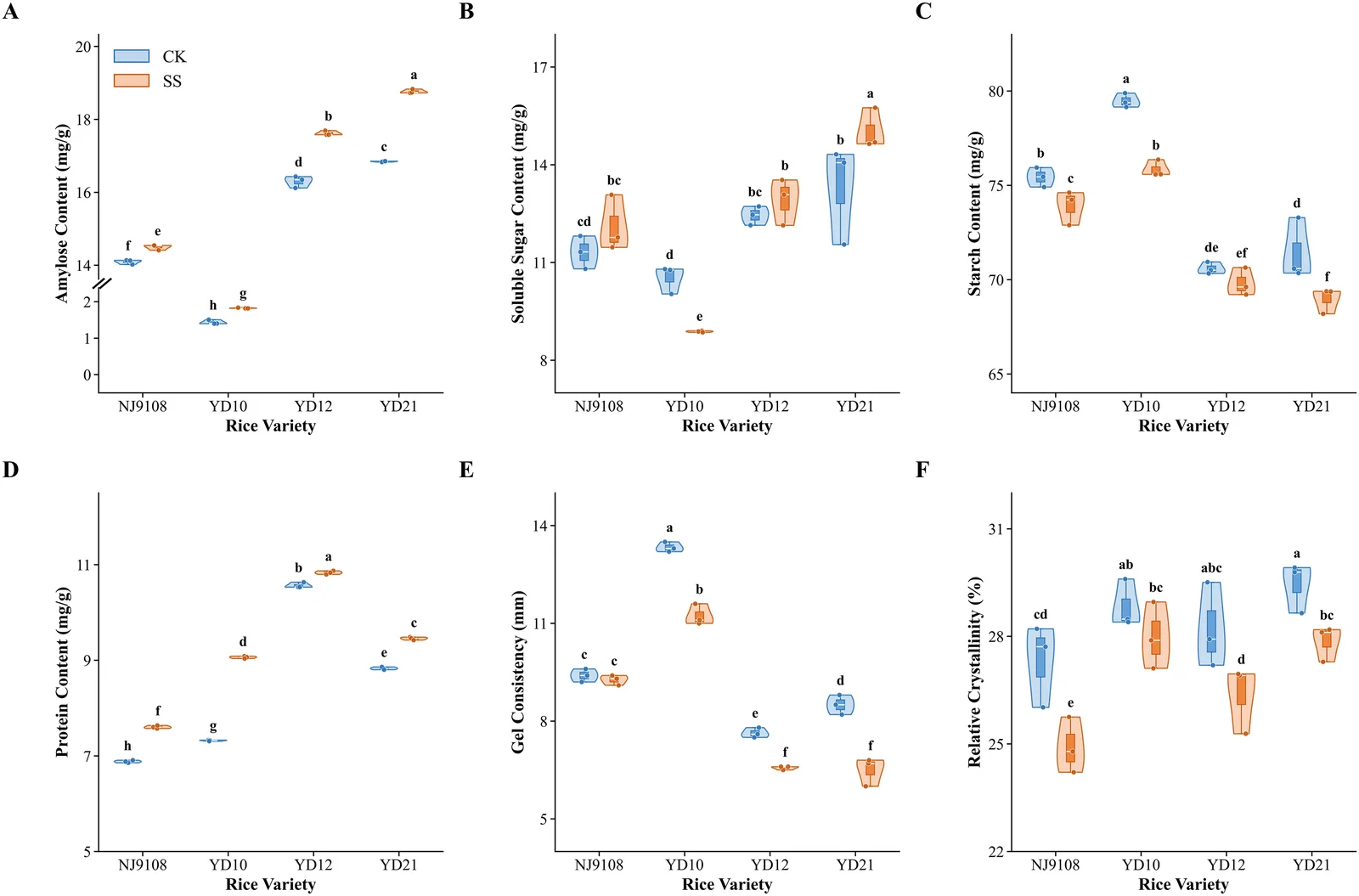
Grain composition and quality parameters under saline-alkali stress. **(A)** Amylose, **(B)** soluble sugar, **(C)** starch, **(D)** protein, **(E)** gel consistency, **(F)** relative crystallinity. CK, control; SS, saline-alkali stress. Letters indicate significant differences at *p < 0.05* (Duncan’s test). n = 3.

Soluble sugar content showed variety-specific responses (**Figure 1B**). Under saline-alkali stress, soluble sugar content increased in Yandao 21, Nanjing 9108, and Yandao 12 but decreased in Yandao 10. Yandao 21 showed the most pronounced increase, from 13.2% to 15.0%, equivalent to an increase of 1.8 percentage points or 13.6% relative to CK. In contrast, Yandao 10 decreased from 10.4% to 8.9%, suggesting weaker carbohydrate adjustment under stress. The active soluble sugar accumulation in tolerant varieties likely reflects osmotic adjustment mechanisms.

Total starch content decreased under saline-alkali stress in all varieties, with significant genotypic differences (**Figure 1C**). Yandao 12 showed the smallest reduction (from 70.0% to 69.0%, −1.4%), while Yandao 10 showed the largest (from 78.8% to 76.0%, −3.6%), indicating substantial variation in starch synthesis stability under stress. The universal starch reduction aligns with the observed inhibition of starch synthesis enzyme activities (Section 3.4).

Total protein content increased significantly in all varieties under stress (*p < 0.01*; **Figure 1D**). Nanjing 9108 and Yandao 10 showed larger increases than Yandao 12 and Yandao 21. This pattern suggests stronger stress-induced nitrogen accumulation in the more sensitive genotypes, whereas the more tolerant varieties maintained a more moderate protein response and a better balance between starch and protein accumulation.

Gel consistency decreased under saline-alkali stress in all varieties (**Figure 1E**), indicating firmer cooked rice texture. Yandao 10 showed the largest reduction (from 13.30 mm to 11.25 mm), while Nanjing 9108 showed no significant change. The decreased gel consistency under stress suggests altered starch swelling and gelation properties, which may affect eating quality.

Relative crystallinity, determined by XRD analysis, decreased significantly under stress in all varieties (*p < 0.05* or *p < 0.01*; **Figure 1F**). Nanjing 9108 showed the largest decrease (from 27.31% to 24.92%), followed by Yandao 12 (from 28.21% to 26.38%). Yandao 10 showed the smallest decrease (from 28.83% to 27.99%), while Yandao 21 maintained the highest absolute crystallinity under stress (from 29.45% to 27.86%), consistent with its superior stability in other quality parameters. The reduction in crystallinity indicates disruption of the ordered structure of starch granules under saline-alkali stress.

### 3.3 Protein fractions

Saline-alkali stress significantly altered protein fraction composition (**Table 2**). Glutelin, the major storage protein, increased under stress in all varieties. Prolamin also generally increased, with Yandao 10 and Nanjing 9108 showing greater increases. Globulin showed the most dramatic changes with strong variety-specific patterns: in Yandao 10, globulin decreased from 3.64 to 3.00 mg/g, whereas in Yandao 12 and Yandao 21, it increased sharply from 0.53 and 0.89 mg/g to 2.09 and 3.27 mg/g, respectively. Albumin changes were relatively small and variety-specific. The distinct globulin response patterns between sensitive and tolerant varieties suggest differential metabolic enzyme reprogramming under stress.

**Table 2:** Effects of saline-alkali stress on protein fractions (mg/g).

Variety	Treatment	Albumin	Globulin	Prolamin	Glutelin
NJ9108	CK	1.57 ± 0.01 d	1.74 ± 0.03 f	3.86 ± 0.07 f	24.76 ± 0.40 g
NJ9108	SS	1.59 ± 0.01 d	2.00 ± 0.02 e	4.12 ± 0.02 d	27.21 ± 0.47 f
YD10	CK	1.57 ± 0.02 d	3.64 ± 0.03 a	2.63 ± 0.02 h	25.67 ± 0.10 g
YD10	SS	1.74 ± 0.01 a	3.00 ± 0.01 c	3.47 ± 0.01 g	31.24 ± 0.60 d
YD12	CK	1.64 ± 0. 01 c	0.53 ± 0.06 h	4.20 ± 0.01 c	34.43 ± 0.54 c
YD12	SS	1.21 ± 0.01 f	2.09 ± 0.05 d	4.36 ± 0.03 a	35.43 ± 0.95 b
YD21	CK	1.47 ± 0.03 e	0.89 ± 0.02 g	3.99 ± 0.04 e	30.06 ± 0.24 e
YD21	SS	1.67 ± 0.01 b	3.27 ± 0.02 b	4.26 ± 0.02 b	36.69 ± 0.50 a

Different letters indicate significant differences at *p* < 0.05 (Duncan’s test).

### 3.4 Starch synthesis enzyme activities

Saline-alkali stress significantly affected the activities of key starch synthesis enzymes during grain filling (**Figure 2**). AGPase activity peaked at 15 DAP in all varieties under control conditions, with Yandao 21 and Yandao 12 showing the highest activities. Under stress, AGPase activity decreased significantly in all varieties, particularly at 15 DAP and 45 DAP. Nanjing 9108 showed the largest reduction, with AGPase activity at 15 DAP decreasing by approximately 41% under stress compared to CK. Yandao 21 exhibited the smallest reduction, maintaining relatively high AGPase activity under stress, suggesting better preservation of starch initiation capacity.

**Figure 2:**
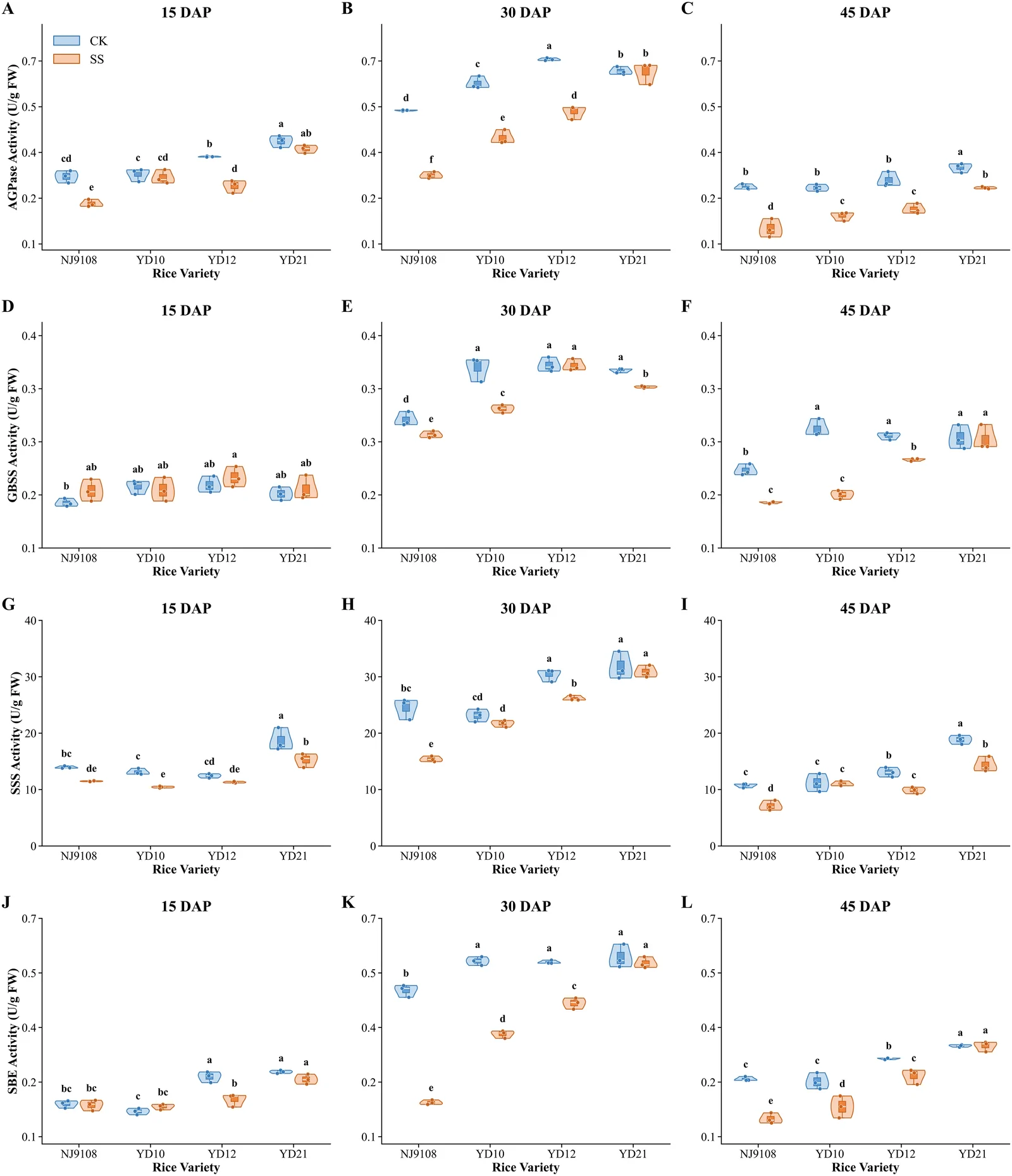
Starch synthesis enzyme activities at 15, 30, and 45 DAP under saline-alkali stress. **(A–L)** AGPase, GBSS, SSS, and SBE at each time point. Letters indicate significant differences at *p < 0.05* (Duncan’s test). n = 3.

GBSS activity, responsible for amylose synthesis, also peaked at 15 DAP. Under saline-alkali stress, GBSS activity decreased in all varieties, with Nanjing 9108 and Yandao 10 showing larger reductions than Yandao 12 and Yandao 21. This pattern is consistent with the observed amylose content changes, where salt-tolerant varieties maintained more stable amylose synthesis.

SSS activity showed the most dramatic response to stress. Under control conditions, Yandao 21 exhibited the highest SSS activity, followed by Yandao 12. Under stress, SSS activity decreased significantly in all varieties, with Nanjing 9108 showing a reduction of over 50% at 15 DAP. Yandao 21 maintained the highest absolute SSS activity under stress, although it still experienced a notable decrease. The severe SSS inhibition in sensitive varieties likely contributes to their greater starch content reduction.

SBE activity peaked at 15 DAP under control conditions. Under stress, SBE activity decreased dramatically in Nanjing 9108 (by approximately 60% at 15 DAP) and Yandao 10 (by approximately 33% at 15 DAP). In contrast, Yandao 21 maintained remarkably stable SBE activity under stress, with almost no change at 15 DAP. This stability in SBE activity likely contributes to Yandao 21’s superior starch quality maintenance under saline-alkali stress. Overall, the enzyme activity results indicate that saline-alkali stress disrupts starch synthesis primarily by inhibiting AGPase, SSS, and SBE activities during the critical mid-filling stage (15 DAP). Salt-tolerant varieties, particularly Yandao 21, exhibited better enzyme activity stability, which correlates with their maintained starch content and quality parameters.

### 3.5 Oxidative stress response

Saline-alkali stress induced significant oxidative stress in rice flag leaves, as evidenced by changes in MDA content and antioxidant enzyme activities (**Figure 3**). MDA content, a marker of lipid peroxidation, increased significantly under stress in all varieties at all measured time points. The increase was most pronounced at 45 DAP, with MDA content approximately doubling under stress compared to CK in all varieties. This indicates that saline-alkali stress causes substantial membrane damage during grain filling, regardless of variety tolerance level.

**Figure 3:**
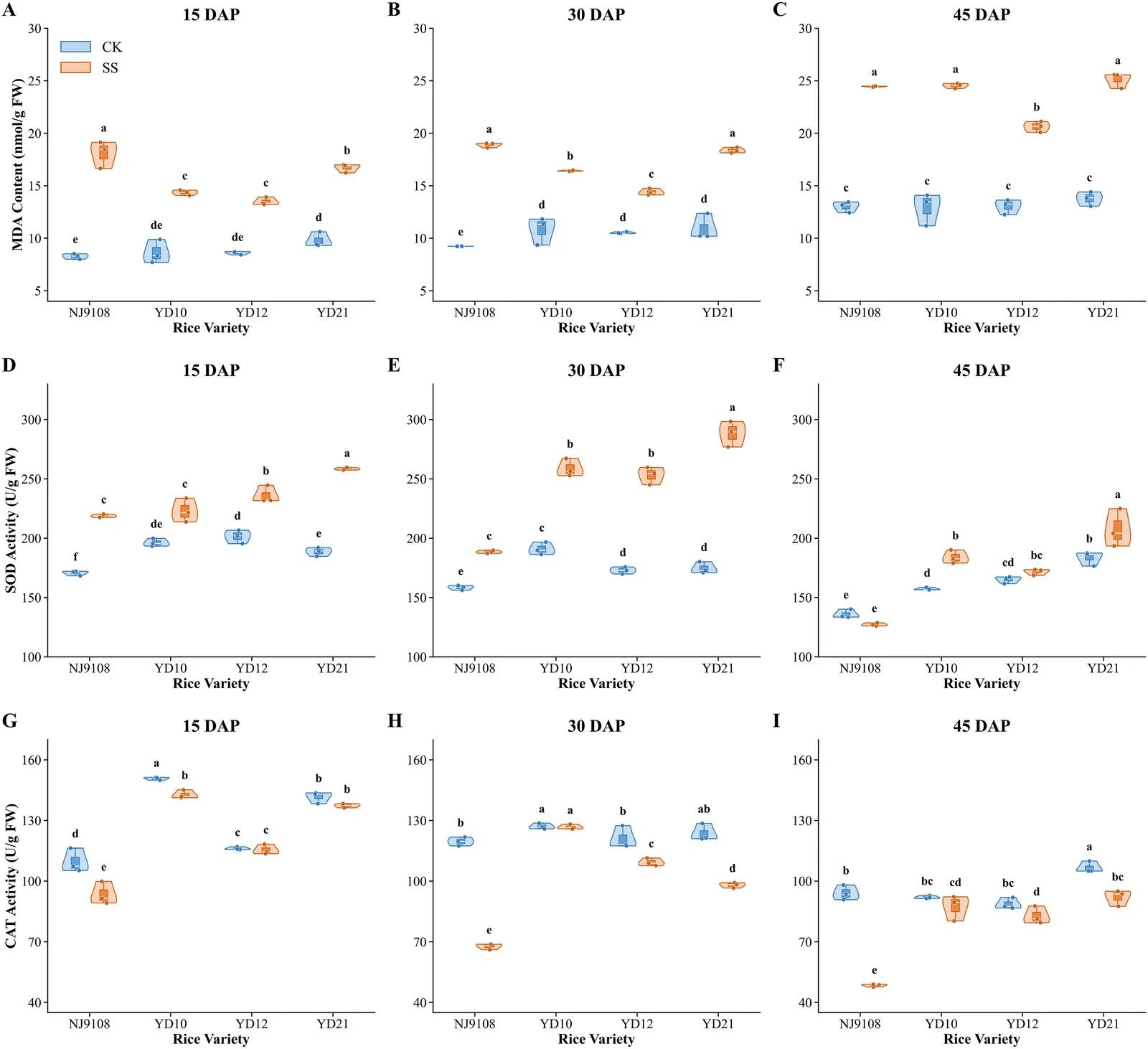
Oxidative stress markers in flag leaves at 15, 30, and 45 DAP under saline-alkali stress. **(A–I)** MDA, SOD, and CAT at each time point. Letters indicate significant differences at *p < 0.05* (Duncan’s test). n = 3.

SOD activity responded differently across varieties. Under stress, SOD activity increased significantly in Yandao 10, Yandao 12, and Yandao 21, particularly at 15 DAP and 30 DAP. Yandao 21 showed the strongest SOD response, with activity increasing by approximately 50% at 15 DAP under stress. In contrast, Nanjing 9108 showed decreased SOD activity at 45 DAP under stress, indicating compromised antioxidant capacity in the salt-sensitive variety during late grain filling.

CAT activity generally decreased under stress, with the most significant reductions observed in Nanjing 9108 at 15 DAP and 45 DAP. Yandao 10 and Yandao 12 also showed decreased CAT activity under stress, while Yandao 21 maintained relatively stable CAT activity compared to the other varieties. These results suggest that salt-tolerant varieties activate SOD-mediated antioxidant defense more effectively under saline-alkali stress, while salt-sensitive varieties suffer from compromised CAT activity and elevated oxidative damage. The differential oxidative stress responses likely contribute to the observed differences in starch synthesis enzyme activities and final grain quality.

### 3.6 Pasting properties

Saline-alkali stress significantly affected pasting properties (**Table 3**). Peak viscosity responses varied: Nanjing 9108 and Yandao 10 increased, Yandao 12 slightly decreased, and Yandao 21 remained stable. Breakdown values increased significantly in Nanjing 9108 and Yandao 10—especially in Yandao 10 (from 654.7 to 993.3 cP)—while Yandao 12 and Yandao 21 showed more moderate increases. Setback values decreased in all varieties, with Yandao 21 showing the largest decrease (from 720.7 to 273.0 cP), while Nanjing 9108 shifted from positive to negative values (from 56.3 to -10.0 cP). Overall, Yandao 12 and Yandao 21 exhibited better stability in pasting parameters under stress, consistent with their superior salt tolerance.

**Table 3:** Effects of saline-alkali stress on pasting properties.

Variety	Treatment	Peak viscosity (cP)	Hot paste viscosity (cP)	Breakdown (cP)	Final viscosity (cP)	Setback (cP)	Pasting time (min)	Pasting temp (°C)
NJ9108	CK	2260.3 ± 28.5 d	1721.7 ± 36.2 e	538.7 ± 54.0 c	2316.7 ± 19.1 f	56.3 ± 33.3 d	6.6 ± 0.8 b	75.8 ± 0.3 bc
NJ9108	SS	2441.0 ± 76.0 b	1774.7 ± 57.1 e	666.3 ± 39.3 b	2431.0 ± 62.2 e	-10.0 ± 27.7 e	6.6 ± 0.0 b	76.8 ± 0.1 bc
YD10	CK	947.0 ± 18.3 f	259.0 ± 48.5 g	654.7 ± 21.2 b	386.7 ± 5.1 h	-560.3 ± 13.8 f	3.6 ± 0.7 a	74.2 ± 0.9 a
YD10	SS	1357.7 ± 19.2 e	364.3 ± 2.1 f	993.3 ± 17.2 a	474.7 ± 9.1 g	-883.0 ± 18.5 g	3.7 ± 0.0 a	74.2 ± 0.5 a
YD12	CK	2340.7 ± 9.1 c	2142.3 ± 17.2 c	198.3 ± 18.4 f	3024.0 ± 40.9 b	683.3 ± 49.9 a	7.0 ± 0.0 c	74.4 ± 0.5 ab
YD12	SS	2254.7 ± 26.6 d	1935.3 ± 26.5 d	319.3 ± 20.6 e	2774.7 ± 42.1 d	520.0 ± 19.5 b	7.0 ± 0.0 c	77.3 ± 0.4 c
YD21	CK	2642.7 ± 42.1 a	2390.7 ± 49.7 a	252.0 ± 59.4 f	3363.3 ± 73.2 a	720.7 ± 38.5 a	7.0 ± 0.0 c	77.3 ± 0.5 c
YD21	SS	2620.7 ± 14.0 a	2232.3 ± 96.8 b	388.3 ± 92.0 d	2893.7 ± 13.9 c	273.0 ± 14.4 c	7.0 ± 0.4 c	77.3 ± 0.9 c

Different letters within the same column indicate significant differences at *p* < 0.05. (Duncan’s test).

### 3.7 Thermal properties

Saline-alkali stress systematically affected thermal properties (**Table 4**). Gelatinization enthalpy (ΔHgel) decreased significantly in all varieties, indicating disruption of starch crystalline structure. Nanjing 9108 and Yandao 10 showed reductions exceeding 50%, while Yandao 21 maintained the highest absolute enthalpy under stress. Retrogradation percentage (%R) decreased dramatically to below 5% in all varieties under stress, far lower than control levels (up to 31%), indicating strong inhibition of starch retrogradation. Retrogradation enthalpy (ΔHret) also decreased substantially. Changes in pasting temperature were relatively small (mostly within 1 °C), suggesting relative stability of starch crystal melting temperature ranges. Calculation of the ΔHgel/crystallinity ratio revealed that under stress, this ratio decreased substantially in Nanjing 9108 (from 0.323 to 0.167, -48.4%) and Yandao 10 (from 0.299 to 0.149, -50.2%), but only moderately in Yandao 12 (from 0.337 to 0.295, -12.5%) and Yandao 21 (from 0.383 to 0.338, -11.7%). The proportionally greater decrease in ΔHgel relative to crystallinity in sensitive varieties indicates that crystal perfection (quality) was more affected than crystal quantity, consistent with more severe disruption of amylopectin branching patterns by SSS and SBE inhibition.

**Table 4:** Effects of saline-alkali stress on thermal properties.

Variety	Treatment	ΔHgel (J/g)	Tp (°C)	To (°C)	Tc (°C)	ΔHret (J/g)	Retrogradation (%)
NJ9108	CK	8.818 ± 0.436 d	72.500 ± 0.100 a	66.800 ± 0.624 b	79.333 ± 0.351 ab	0.380 ± 0.06 b	4.333 ± 0.577 c
NJ9108	SS	4.160 ± 0.029 b	72.900 ± 0.300 a	67.667 ± 0.404 a	79.133 ± 0.404 ab	0.070 ± 0.01 b	1.667 ± 0.577 c
YD10	CK	8.617 ± 0.238 bc	71.867 ± 0.208 b	65.467 ± 0.231 c	79.700 ± 0.529 a	1.839 ± 0.41 a	21.333 ± 4.619 ab
YD10	SS	4.175 ± 0.097 d	71.267 ± 0.058 c	66.067 ± 0.208 c	78.167 ± 0.153 c	0.154 ± 0.02 b	3.667 ± 0.577 c
YD12	CK	9.494 ± 1.283 b	71.500 ± 0.265 bc	65.367 ± 0.208 c	78.667 ± 0.681 bc	2.857 ± 1.53 a	31.333 ± 18.903 a
YD12	SS	7.779 ± 0.183 c	71.333 ± 0.153 c	64.400 ± 0.529 d	78.567 ± 0.208 bc	0.202 ± 0.02 b	2.667 ± 0.577 c
YD21	CK	11.293 ± 0.222 a	71.667 ± 0.306 bc	65.700 ± 0.173 c	79.300 ± 0.458 ab	1.978 ± 0.83 a	17.667 ± 7.506 b
YD21	SS	9.405 ± 0.066 b	71.800 ± 0.436 b	66.033 ± 0.473 c	79.000 ± 0.889 ab	0.137 ± 0.02 b	1.333 ± 0.577 c

Different letters within the same column indicate significant differences at *p* < 0.05. (Duncan’s test).

### 3.8 Crystalline structure

XRD analysis revealed distinct differences in starch crystalline structure among varieties and treatments (**Figure 4**). All samples exhibited typical A-type crystalline patterns with characteristic diffraction peaks at approximately 15°, 17°, 18°, and 23° 2θ. Under saline-alkali stress, relative crystallinity decreased significantly in all varieties. Nanjing 9108 showed the largest decrease in relative crystallinity (from 27.31% to 24.92%), followed by Yandao 12 (from 28.21% to 26.38%). Yandao 10 and Yandao 21 showed smaller but still significant decreases.

**Figure 4:**
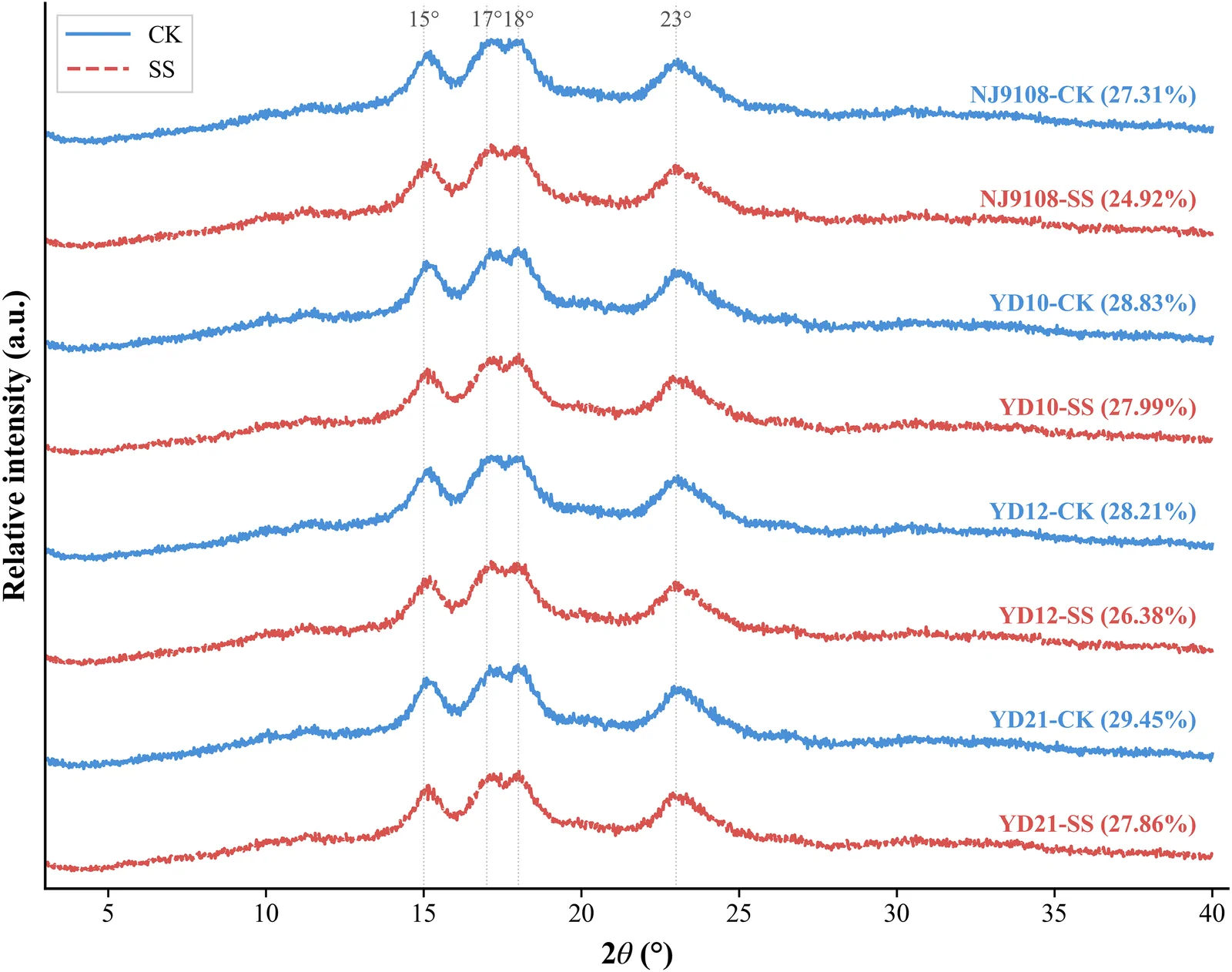
XRD patterns of starch from four rice varieties. Relative crystallinity (%) is annotated next to each curve. CK (solid lines), control; SS (dashed lines), saline-alkali stress. A-type crystalline peaks at approximately 15, 17, 18, and 23 2theta.

The reduction in relative crystallinity under stress indicates disruption of the ordered crystalline structure of starch granules. This structural change is consistent with the observed decreases in gelatinization enthalpy and retrogradation parameters, suggesting that saline-alkali stress produces starch with lower crystallinity and more amorphous regions. The greater crystallinity reduction in sensitive varieties correlates with their more severe starch synthesis enzyme inhibition and quality deterioration. The maintained crystallinity in Yandao 21 aligns with its superior thermal stability and pasting property maintenance.

### 3.9 Starch granule morphology

SEM observation of starch granule morphology revealed treatment- and variety-specific differences (**Figure 5**). Under control conditions, all varieties exhibited typical polyhedral starch granules with smooth surfaces and well-defined edges. Under saline-alkali stress, starch granules displayed varying degrees of morphological alteration. In Nanjing 9108 and Yandao 10, some granules appeared swollen, with less distinct edges and rougher surfaces. In contrast, Yandao 12 and Yandao 21 maintained relatively better-preserved granule morphology under stress, although minor surface irregularities were still observable.

**Figure 5:**
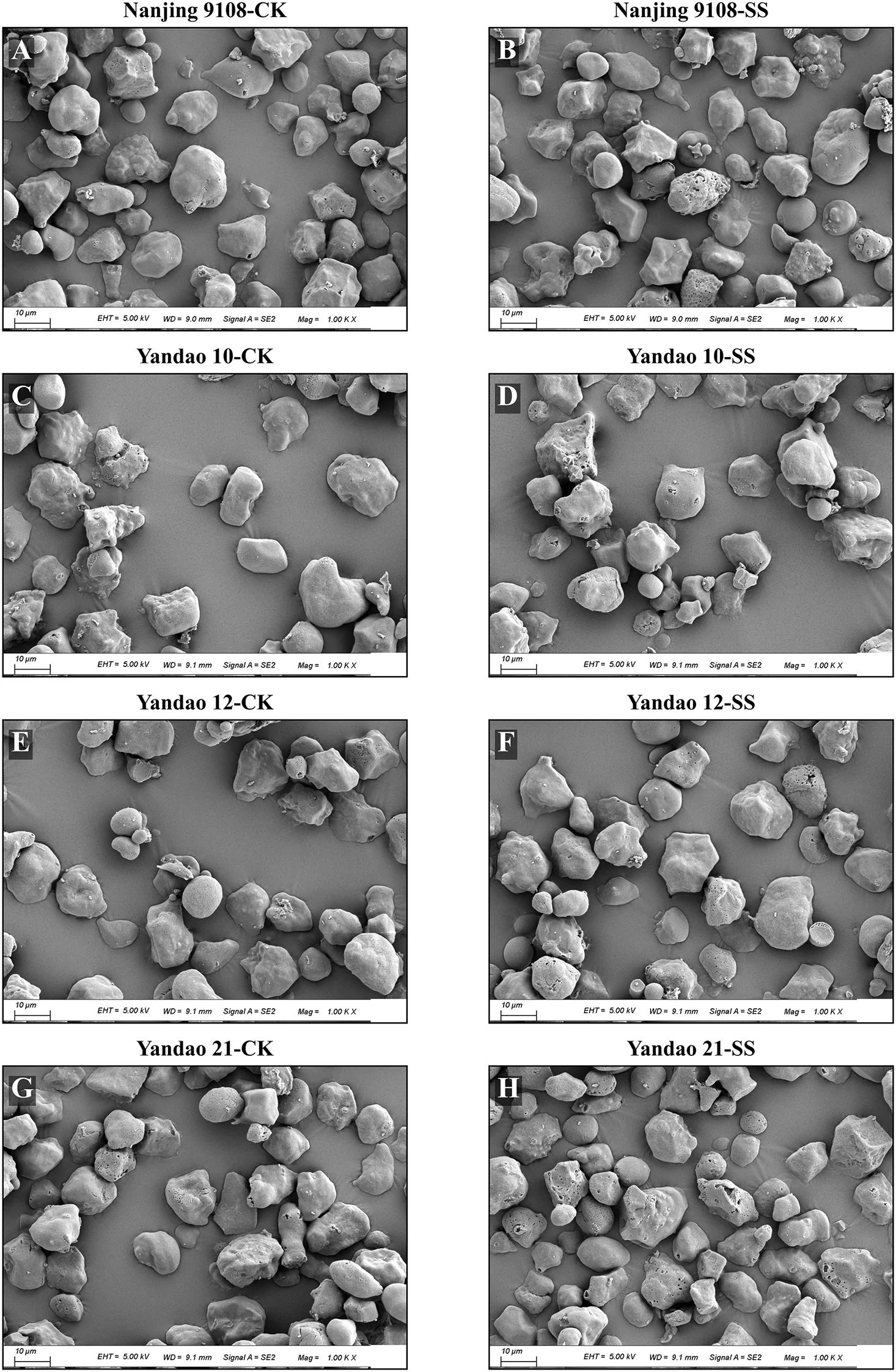
SEM images of starch granules from four rice varieties. **(A)** NJ9108 CK, **(B)** NJ9108 SS, **(C)** YD10 CK, **(D)** YD10 SS, **(E)** YD12 CK, **(F)** YD12 SS, **(G)** YD21 CK, **(H)** YD21 SS. Scale bar = 10 µm. CK, control; SS, saline-alkali stress.

These morphological changes are consistent with the observed reductions in crystallinity and the alterations in pasting properties. The disrupted granule surface structure in sensitive varieties likely contributes to their higher breakdown values during RVA analysis, as damaged granules are more prone to rupture upon heating. The relatively intact granule morphology in Yandao 21 aligns with its stable pasting properties and maintained eating quality under stress.

### 3.10 Correlation network analysis

Correlation network analysis visualized interrelationships among grain quality, enzyme activities, and oxidative stress markers (**Figure 6**). The network comprised 13 indicators connected by 23 significant edges (17 positive, 6 negative; |r| > 0.6, *p* < 0.05). Crystallinity, AGPase, SSS, and SBE emerged as hub nodes (degree = 5 each), indicating their central roles in linking grain quality, starch metabolism, and oxidative stress. Positive correlations predominated among starch synthesis enzymes and quality parameters, while negative correlations linked MDA to enzyme activities, confirming that oxidative damage is closely associated with starch biosynthesis inhibition.

**Figure 6:**
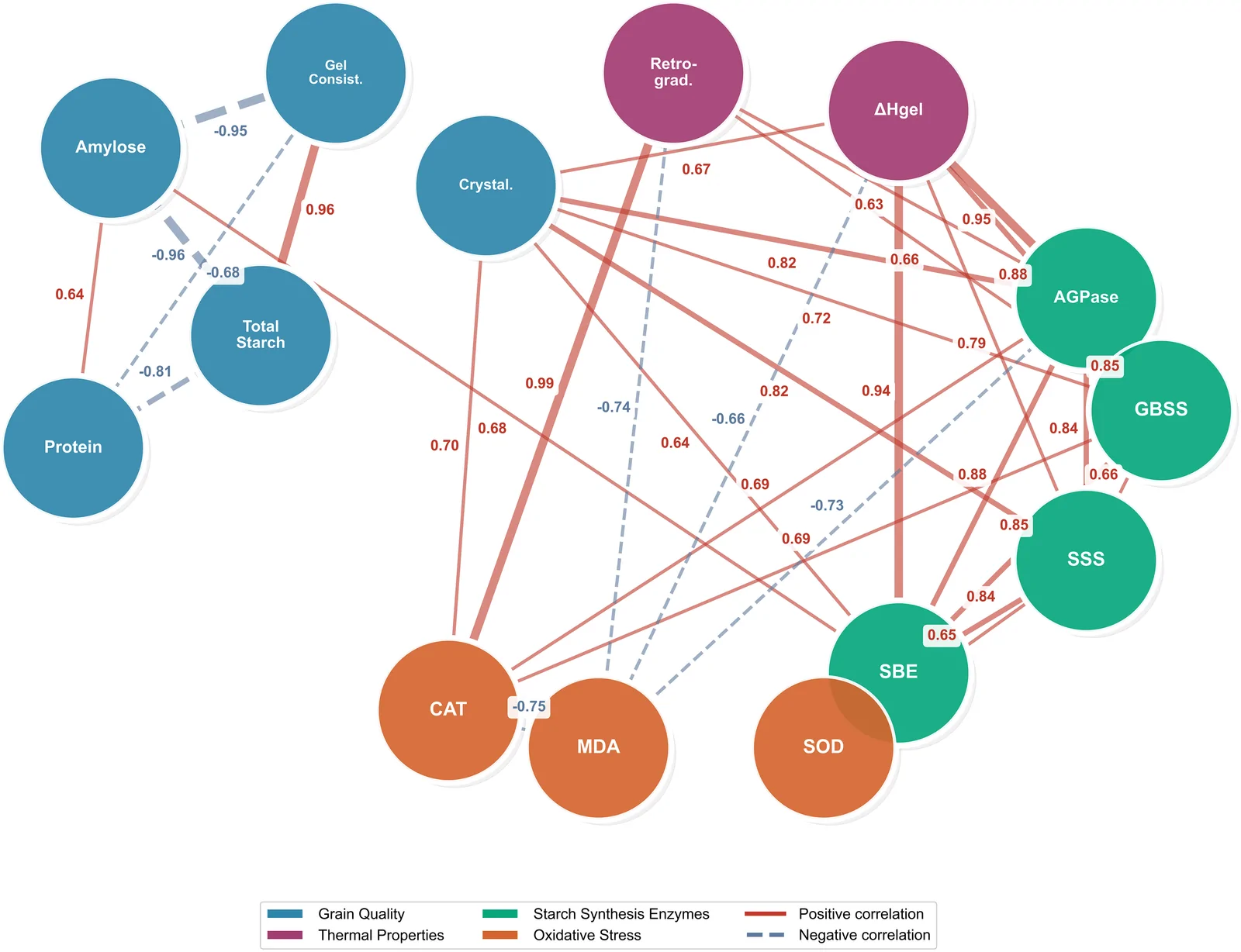
Correlation network of quality, thermal, enzyme, and oxidative stress parameters. Blue, purple, green, and orange nodes: quality, thermal properties, enzyme, and oxidative stress indicators. Red and blue lines: positive and negative correlations (|r| ≥ 0.6, *p < 0.05*).

The correlation network results demonstrate that saline-alkali stress induces coordinated changes across multiple quality and physiological parameters, with starch synthesis enzymes serving as critical intermediaries linking oxidative damage to grain quality deterioration.

## 4 Discussion

### 4.1 Carbon-nitrogen metabolic imbalance underlies quality deterioration

The present study demonstrated that coastal saline-alkali stress (3.0‰ soil salt) consistently increased amylose content while reducing total starch across all four rice varieties, indicating a fundamental perturbation of carbon allocation within the developing endosperm. This apparent paradox—concomitant amylose enrichment and total starch depletion—reflects a rechanneling of carbon flux away from starch biosynthesis and toward soluble sugars and other osmoprotectants, a widely documented adaptive strategy under osmotic stress (Guo et al., 2023a). Notably, the salt-tolerant variety YD21 exhibited the most pronounced accumulation of soluble sugars (+13.6%), whereas the salt-sensitive NJ9108 showed a comparatively modest response, suggesting that the capacity to redirect carbon into compatible solutes is a hallmark of tolerance rather than susceptibility. These observations are consistent with recent reports that salinity increases amylose content and decreases total starch in a genotype-dependent manner (Sangwongchai et al., 2022; Li et al., 2023; Zhang et al., 2022; Sangwongchai et al., 2025), and they extend those findings by linking the magnitude of soluble sugar accumulation to overall tolerance. Recent metabolomic evidence further supports this interpretation, demonstrating that salt stress profoundly reprograms carbon metabolism in rice, including the upregulation of sucrose synthase and amylase activities and the redistribution of non-structural carbohydrates (Jin et al., 2024; Wang et al., 2024).

The concurrent increase in total protein content under stress further supports the notion of a carbon-nitrogen metabolic imbalance. Under saline-alkali conditions, the inhibition of starch biosynthesis may free up carbon skeletons and energy that are rerouted into nitrogen assimilation and protein synthesis, as has been reported in rice subjected to salt-alkaline stress (Guo et al., 2023a, Guo et al., 2023b). This shift in metabolic partitioning has direct implications for eating and cooking quality: elevated amylose generally increases grain hardness and reduces stickiness, while higher protein content can strengthen the protein matrix surrounding starch granules, restricting water absorption during cooking and altering texture (Guo et al., 2023b; Hu et al., 2024). The genotype-specific protein responses observed in this study suggest that the balance between carbon and nitrogen metabolism is a key determinant of quality stability under stress. These findings resonate with recent evidence that nitrogen availability and stress-induced metabolic reprogramming jointly modulate the eating quality of rice (Guo et al., 2023; Hu et al., 2024), highlighting the importance of managing carbon-nitrogen partitioning as a breeding target. It should be noted that the evidence for carbon-nitrogen metabolic imbalance is indirect, being inferred from changes in starch and protein fractions rather than from direct metabolite measurements; future metabolomic analyses would provide more direct confirmation.

### 4.2 Starch biosynthesis enzyme inhibition drives structural and functional changes

The activities of all four major starch synthesis enzymes—AGPase, GBSS, SSS, and SBE—were significantly inhibited under saline-alkali stress, with the most pronounced suppression occurring at 15 days after pollination (DAP), a critical window for starch granule initiation and amylopectin branching (Lee et al., 2021; Praphasanobol et al., 2025). This temporal pattern is of considerable mechanistic significance: AGPase catalyzes the rate-limiting step of ADP-glucose formation, while SSS and SBE jointly determine the branch chain length and crystalline structure of amylopectin. Their concurrent inhibition at 15 DAP would be expected to reduce the synthesis of short-chain branches, impair granule packing, and ultimately compromise the structural integrity of the starch granule (Praphasanobol et al., 2025; Ma et al., 2026). GBSS, responsible for amylose synthesis, was also inhibited, yet amylose content paradoxically increased under stress, likely because the relative inhibition of SSS and SBE was more severe, leading to a higher proportion of amylose within a reduced total starch pool (Zheng et al., 2024).

The functional consequences of these enzymatic and structural changes were evident in the thermal and pasting properties of the starch. Gel consistency, gelatinization enthalpy (ΔHgel), and retrogradation all decreased under stress, consistent with the formation of starch granules with lower crystalline order and reduced capacity for molecular reassociation (Ma et al., 2026; Wang et al., 2023, 2015). XRD analysis confirmed a significant reduction in relative crystallinity in all varieties, with the greatest decrease observed in the salt-sensitive NJ9108 (from 27.31% to 24.92%), while YD21 maintained the highest absolute crystallinity under stress (from 29.45% to 27.86%). Importantly, YD21 retained 83% of its gelatinization enthalpy under stress (stress tolerance index, STI = 0.83) and exhibited the lowest retrogradation, indicating that its starch maintained a higher degree of crystalline perfection. This suggests that YD21 preserves the coordinated activity of SSS and SBE to a greater extent than the sensitive varieties, sustaining amylopectin branching and granule organization. These observations align with recent evidence that salt stress disrupts starch crystalline structure and that the extent of structural disruption correlates with the severity of enzymatic inhibition (Zhang et al., 2022; Sangwongchai et al., 2025; Jin et al., 2024). Collectively, the data indicate that the differential retention of starch synthesis enzyme activity, particularly during the early grain-filling phase, is a primary determinant of genotype-specific starch quality stability under saline-alkali stress.

### 4.3 Oxidative stress as a critical hub linking saline-alkali stress to quality deterioration

Oxidative stress emerged as a central hub linking saline-alkali stress perception to grain quality deterioration. Malondialdehyde (MDA), a reliable marker of lipid peroxidation and membrane damage, accumulated in all varieties under stress, but the magnitude of accumulation was substantially lower in tolerant genotypes. Concurrently, the antioxidant defense system responded in a genotype-dependent manner: superoxide dismutase (SOD) was strongly activated in tolerant varieties—particularly YD21, which showed an approximately 50% increase in SOD activity at 15 DAP—whereas catalase (CAT) activity declined sharply in the sensitive NJ9108. This divergence indicates that tolerant genotypes maintain a more effective ROS-scavenging capacity during grain filling, thereby limiting oxidative damage to the developing endosperm (Tavu and Redillas, 2025; Du et al., 2025). Recent studies have demonstrated that exogenous application of uniconazole and melatonin can mitigate salt-induced oxidative damage by enhancing SOD and CAT activities, providing direct experimental support for the notion that antioxidant capacity is a critical determinant of quality maintenance under salt stress (Du et al., 2025; Ubaidillah et al., 2024; Alharbi et al., 2022).

Correlation network analysis further substantiated the pivotal mediating role of oxidative stress. AGPase, SSS, and SBE were identified as hub nodes (degree = 5 each), tightly connecting oxidative stress markers (MDA, SOD, CAT) to multiple quality traits. This suggests that oxidative damage is closely associated with starch synthesis enzyme inhibition, consistent with the hypothesis that oxidative damage exerts its deleterious effects on grain quality at least in part through the inhibition of starch synthesis enzymes. This mechanistic linkage is biologically plausible: ROS can directly oxidize and modify enzyme proteins, impairing their catalytic function, and the developing endosperm—with its high metabolic activity and limited antioxidant buffering capacity—is particularly vulnerable to such damage (Tavu and Redillas, 2025). Exogenous application of ROS-scavenging agents, including melatonin and uniconazole, has been shown to mitigate salt-induced oxidative damage and partially restore starch biosynthesis, providing strong causal support for the interpretation of our correlation findings (Du et al., 2025; Ubaidillah et al., 2024; Cheng et al., 2025). From a mechanistic perspective, the oxidative robustness of starch synthesis enzymes, together with the upstream antioxidant systems that protect them, may constitute a critical physiological target for improving quality stability under saline-alkali stress.

### 4.4 Protein fraction remodeling reflects genotype-specific adaptation strategies

The increase in total protein content under saline-alkali stress, accompanied by variety-specific remodeling of protein fractions, points to a dynamic reorganization of nitrogen metabolism that may serve both structural and protective functions. In rice, storage proteins—predominantly glutelin, prolamin, and globulin—form a matrix around starch granules that directly influences grain hardness, pasting viscosity, and cooked rice texture (Guo et al., 2023b). Stress-induced protein accumulation can therefore be interpreted as a dual response: it reflects the rerouting of carbon and nitrogen assimilates away from starch biosynthesis, and it may also contribute to stress adaptation through the synthesis of protective proteins and the reinforcement of the protein-starch matrix (Hu et al., 2024). In rice endosperm, globulin has been associated with stress-responsive accumulation, mineral sequestration under ionic stress, and structural interactions with starch granules that influence pasting properties (Guo et al., 2023b; Hu et al., 2024). The sharp increase in globulin in tolerant varieties (Yandao 12 and Yandao 21) may therefore serve dual functions: osmoprotection through mineral binding and structural reinforcement of the protein-starch matrix, both of which could contribute to the superior quality stability observed in these genotypes. However, the precise functional roles of these globulin changes warrant further investigation through proteomic and functional genomic approaches.

The genotype-specific protein responses observed here are particularly noteworthy. Tolerant varieties exhibited a more balanced protein accumulation pattern that preserved starch granule integrity and supported acceptable gelatinization behavior, whereas the sensitive NJ9108 displayed protein profiles associated with inferior eating quality. Most strikingly, globulin showed the most dramatic genotype-specific changes: in YD10, globulin decreased from 3.64 to 3.00 mg/g, whereas in YD12 and YD21, it increased sharply from 0.53 and 0.89 mg/g to 2.09 and 3.27 mg/g, respectively. This profound divergence in globulin response may reflect fundamental differences in metabolic enzyme reprogramming strategies between sensitive and tolerant varieties, potentially linked to differential stress signaling and nitrogen remobilization patterns. These findings resonate with recent evidence that the OsGAPC3 mutation improves both nutritional and processing quality by modulating protein composition, underscoring the tight coupling between protein remodeling and overall quality (Peng et al., 2024). Notably, the specific protein fraction changes observed here appear to be connected to pasting property stability: the higher globulin content in tolerant varieties (Yandao 12, Yandao 21) may strengthen the protein matrix surrounding starch granules, limiting granule swelling and contributing to their more stable pasting profiles (**Table 3**), whereas the greater glutelin increase in Nanjing 9108 may partly contribute to its more pronounced viscosity breakdown. Accordingly, our analysis of the interplay between nitrogen metabolism and starch biosynthesis suggests that protein fraction composition must be considered alongside starch-related traits as an important dimension for ensuring quality stability under saline-alkali stress (Guo et al., 2023b; Hu et al., 2024).

### 4.5 Integrated mechanistic framework and study limitations

By integrating the analyses across all of the above dimensions, the present study delineates a physiological cascade through which saline-alkali stress is associated with rice grain quality deterioration: oxidative stress induced by saline-alkali conditions (MDA accumulation, SOD/CAT imbalance) → inhibition of key starch synthesis enzymes (AGPase, SSS, SBE) in the developing endosperm → disruption of carbon-nitrogen metabolic balance and impairment of the ordered crystalline structure of starch granules (reduced gel consistency, gelatinization enthalpy, and crystallinity) → ultimate deterioration of eating and cooking quality. By comparing four varieties with contrasting salt tolerance as physiological models, we further established that tolerant varieties—particularly YD21—possess significant advantages in ROS-scavenging capacity, early grain-filling enzyme activity maintenance, and carbon-nitrogen metabolic balance. It should be emphasized that the present study does not aim to screen or recommend specific varieties for breeding; rather, these four genotypes serve as a comparative framework for dissecting the differential physiological strategies that underlie quality stability under saline-alkali stress.

This comprehensive physiological framework provides an important theoretical foundation for subsequent molecular dissection. From a long-term perspective, identifying the key genetic loci that regulate the enzymatic robustness and antioxidant defense systems described above through genomic or transcriptomic approaches—ideally using larger mapping populations—will be a critical direction for future genetic improvement (Wang et al., 2025; Fernando et al., 2025).

Several limitations of this study should nevertheless be acknowledged. First, the experiment was conducted over a single growing season at one site, and the magnitude of stress responses may vary with seasonal climatic and edaphic conditions; multi-year, multi-location validation is warranted. Second, enzyme activity and oxidative stress measurements were conducted at the bulk endosperm level, without resolving the spatial heterogeneity of stress responses within the grain. Third, although correlation network analysis provides clear target-oriented clues, it cannot directly establish causality; future functional validation through gene editing or transgenic approaches targeting the identified hub enzymes (e.g., AGPase, SSS) will be essential to confirm the mechanistic causality (Ahmad et al., 2022; Du et al., 2025; Ubaidillah et al., 2024). Additionally, the absence of fertilizer application, while eliminating confounding effects, may limit the generalizability of the results to fertilized field conditions where nutrient availability could modulate stress responses. Furthermore, the three sampling time points (15, 30, 45 DAP) provide insufficient temporal resolution to capture the dynamics of starch granule initiation between 15 and 30 DAP; finer sampling intervals (e.g., 10, 20 DAP) would better capture the critical window of starch granule formation. Elucidating these physiological pathways and their underlying genetic mechanisms will be critical for translating the present findings into practical strategies for stress-resilient and quality-stable rice production in coastal saline-alkali regions.

## 5 Conclusions

This study provides systematic evidence that coastal saline-alkali stress compromises rice grain quality through a coordinated physiological cascade involving oxidative stress, starch synthesis enzyme inhibition, and structural disruption of starch granules. Salt-tolerant genotypes (Yandao 12 and Yandao 21) maintained higher enzyme activities, more effective ROS scavenging, and better preservation of crystalline structure, whereas Nanjing 9108 suffered the most severe quality deterioration. Correlation network analysis identified AGPase, SSS, and SBE as central hub nodes, providing systematic evidence consistent with the hypothesis that oxidative stress contributes to grain quality deterioration at least in part through the inhibition of starch biosynthesis enzymes.

These findings highlight starch synthesis enzymes and oxidative stress defense systems as priority targets for genetic improvement. Future studies should integrate transcriptomic and proteomic approaches and employ ROS scavengers or gene editing to establish causal relationships definitively.

## Data Availability

The original contributions presented in the study are included in the article/supplementary material. Further inquiries can be directed to the corresponding authors.
